# Radiation knowledge, risk literacy, and concern about heritable effects: a cross-sectional study in Fukushima and the Tokyo metropolitan area

**DOI:** 10.3389/fpubh.2026.1977741

**Published:** 2026-10-14

**Authors:** Yuya Kashiwazaki, Varsha Hande, Makiko Orita, Kyoka Majima, Hitomi Matsunaga, Noboru Takamura

**Affiliations:** Department of Disaster Resilience and Science, Nagasaki University Graduate School of Biomedical Sciences, Nagasaki, Japan

**Keywords:** knowledge, mediation analysis, radiation risk perception, risk communication, risk literacy

## Abstract

**Inroduction:**

Public concern persists about the heritable effects of radiation following the Fukushima Daiichi Nuclear Power Station accident, despite scientific consensus indicating no detectable increase in such effects. This study examined how radiation-related knowledge and multidimensional risk literacy relate to concern about heritable effects, and explored the cognitive mechanisms underlying this concern, among residents of Fukushima Prefecture and the Tokyo metropolitan area (the seven prefectures of the Kanto region).

**Methods:**

A web-based cross-sectional survey was conducted in September 2023 among 1,529 residents of Fukushima Prefecture and the Tokyo metropolitan area. Radiation-related knowledge and risk literacy were assessed using five structured items and a validated 24-item scale comprising six subscales, respectively. Hierarchical logistic regression and exploratory statistical mediation analyses were used to identify independent predictors of concern and to examine the mediating role of risk literacy.

**Results:**

Fukushima residents demonstrated higher radiation-related knowledge, whereas Tokyo residents reported greater concern about heritable effects. In the multivariable model, higher knowledge (odds ratio = 0.77, 95% confidence interval: 0.69–0.85; not significant when the two items closely paralleling the outcome were excluded) and higher scores on several risk literacy subscales, most notably cognitive bias awareness, were independently associated with lower concern. This residence–concern association was somewhat attenuated but persisted after adjustment for these factors (odds ratio = 1.42, compared with 1.53 before adjustment). Mediation analysis indicated that approximately 29% (95% CI [20.1%, 42.2%]) of the association between knowledge and concern was statistically mediated by risk literacy, with the remainder representing an association between knowledge and concern independent of risk literacy.

**Discussion:**

Radiation-related knowledge and multidimensional risk literacy were independently associated with lower concern about heritable effects, and were associated with part of the geographical disparity in concern between Fukushima and Tokyo residents. Because these factors act as complementary rather than interchangeable determinants, reducing unwarranted concern may require not only correcting factual misconceptions but also cultivating public capacity for structured risk reasoning, particularly in regions beyond the directly affected area.

## 1 Introduction

Despite consistent scientific consensus indicating no detectable increase in heritable health effects among the general public, concern about the heritable consequences of radiation exposure has persisted long after nuclear accidents (1–3). This persistent gap between the evidence and the level of concern is of public health interest: concern that substantially exceeds what the evidence supports may itself carry a psychosocial cost, independent of any change in the underlying risk. This study adopts the normative premise, consistent with current scientific consensus, that concern substantially exceeding the evidence for detectable risk may be considered unwarranted, while recognizing that the absence of a detectable increase is not equivalent to an absence of risk. This gap between scientific evaluation and public understanding represents a critical challenge for post-disaster risk communication and public health policy. The Fukushima Daiichi Nuclear Power Station (FDNPS) accident in March 2011, triggered by the Great East Japan Earthquake, provides a salient context in which to examine this phenomenon. The FDNPS accident released radionuclides, including ^131^I and ^137^Cs, in quantities estimated to be 10–20% of those released during the Chernobyl accident (2), and has since generated sustained public concern about heritable effects on future generations—despite repeated affirmations that no such increase in health effects has been observed (1–3). This persistent concern has been documented even among non-evacuee residents living outside directly contaminated areas (4).

Numerous radiation risk communication initiatives in Fukushima Prefecture have successfully led to a decline in risk perception among local residents (5–7). Nonetheless, high levels of anxiety and risk perception about radiation persist among residents living outside Fukushima, especially in urban areas such as Tokyo, where opportunities for direct engagement with radiation experts are limited (8). A series of surveys conducted repeatedly between 2017 and 2023 consistently found that residents of the Tokyo metropolitan area perceived higher health risks associated with Fukushima than Fukushima residents themselves, suggesting that geographical factors and differences in disaster experience may influence risk perception (9, 10). This regional disparity may be partly attributable to differential access to domain-specific knowledge: Fukushima residents, through sustained local risk communication, have had greater opportunity to acquire accurate knowledge of radiation health effects—including the absence of confirmed heritable effects among the offspring of atomic bomb survivors (1)—than their Tokyo counterparts. Within Fukushima itself, health literacy has similarly been shown to relate to radiation anxiety, with associations differing between evacuated and non-evacuated areas (11), suggesting that cognitive and informational resources beyond simple exposure to risk communication may shape regional patterns of concern.

Nevertheless, factual knowledge alone does not fully account for these regional differences. Previous research has shown that while geographical disparities in perception are partially mediated by knowledge, approximately 40% of individuals with accurate knowledge still perceive a high level of heritable risk (12). This limited explanatory power of knowledge is consistent with critiques of the knowledge-deficit model, which posits that providing scientific information alone is sufficient to correct public misperceptions; in reality, the gap between expert and lay risk perception cannot be attributed simply to differences in knowledge (13). Individual and contextual factors, rather than knowledge per se, often act as amplifiers of risk perception (14). These findings suggest that cognitive factors beyond factual knowledge play a significant role in shaping radiation concerns, pointing to the relevance of risk literacy as a complementary explanatory construct.

Risk literacy—the ability to comprehend, interpret, and apply risk-related information to informed decision-making—offers a promising framework for understanding this residual concern. Unlike health literacy, which is broadly defined as the knowledge, motivation, and competencies to access, understand, appraise, and apply health information to make decisions concerning one’s own health (15, 16), risk literacy involves the capacity to navigate scientific uncertainty and evaluate trade-offs. Recent work has begun to examine health literacy in relation to knowledge of radiation’s genetic effects in Fukushima (17), but risk literacy specifically remains comparatively underexamined. Conceptually, risk literacy can be understood as the capacity to interpret and apply factual knowledge within a given risk context, positioning it as a potential mediator between knowledge acquisition and risk perception. Despite its potential relevance in post-disaster contexts, empirical evidence directly linking radiation-related knowledge, risk literacy, and individual risk perception remains limited in Japan.

This study aimed to elucidate the relationship between knowledge and risk literacy regarding radiation risk and their influence on the perception of radiation effects among residents of Fukushima Prefecture and the Tokyo metropolitan area. Specifically, it seeks to (1) compare levels of radiation-related knowledge and risk literacy between the two populations, and (2) examine how these cognitive factors, alongside sociodemographic variables, contribute to perceptions about heritable effects of radiation.

Based on the intensive risk communication in Fukushima Prefecture after the FDNPS accident, we hypothesize that residents of Fukushima possess higher radiation-related knowledge than residents of the Tokyo metropolitan area. Furthermore, we hypothesize that higher levels of risk literacy are independently associated with lower concern, and we explore, in an exploratory statistical mediation framework, whether risk literacy accounts for part of the association between knowledge and concern. Because the cross-sectional design cannot establish temporal ordering, this analysis is treated as hypothesis-generating rather than confirmatory. Additionally, we conduct an exploratory analysis of whether the contribution of these cognitive factors differs between the two regions, given that Tokyo residents may rely more heavily on individual knowledge and literacy due to limited opportunities for direct engagement with radiation issues. By clarifying these associations, the findings will contribute to improving public communication strategies for radiation risk and promoting more scientifically literate and resilient communities nationwide.

This study extends prior work in two respects. First, whereas Kashiwazaki et al. (12) demonstrated that factual knowledge partially mediates the regional gap in radiation risk perception, and Hirota et al. (18) examined the association between knowledge and heritable-effect anxiety within a single region (Fukushima), the present study incorporates a validated multidimensional risk literacy framework alongside knowledge and directly compares Fukushima and Kanto residents within a unified analytic model. Second, by decomposing the association into knowledge-specific and risk-literacy-specific components through exploratory statistical mediation, this study tests whether cognitive factors beyond factual recall contribute independently to explaining regional differences in concern, a question not addressed in either prior study.

## 2 Materials and methods

### 2.1 Study design and participants

A web-based cross-sectional survey was conducted between 8 and 16 September 2023. The questionnaire was administered by Cross Marketing Inc. (Tokyo, Japan), a professional survey firm managing a large nationwide Internet panel in Japan. The provider’s standard data-cleaning protocol, applied prior to data delivery, excludes respondents exhibiting excessively short completion times or straight-lining on scale items. Potential respondents were individuals aged 20–69 years who were registered residents of Fukushima Prefecture or the Tokyo metropolitan area, including Gunma, Tochigi, Ibaraki, Saitama, Chiba, Tokyo, and Kanagawa Prefectures.

To ensure population representativeness, recruitment was conducted using quota sampling according to the demographic profiles of each region. The target distributions for gender and age were as follows: in Fukushima Prefecture, 51% male and 49% female, with 14% in their 20s, 17% in their 30s, 21% in their 40s, 21% in their 50s, and 26% in their 60s; in the Tokyo metropolitan area, 51% male and 49% female, with 16% in their 20s, 19% in their 30s, 25% in their 40s, 22% in their 50s, and 18% in their 60s. Because responses to key sociodemographic and outcome items were mandatory within the online survey platform, no missing data occurred for these variables. Household annual income was the only variable for which non-response was permitted, via a ‘prefer not to answer’ option, resulting in data for 1,214 of 1,529 participants (79.4%); this variable was therefore examined separately in a sensitivity analysis (see below) rather than included in the primary multivariable models. Selection bias was mitigated by recruiting participants from a large Internet panel with broad demographic coverage and applying identical eligibility criteria across regions. With 630 events (participants reporting concern) and 15 parameters in the final multivariable model, the events-per-parameter ratio was 42:1, well above the conventional minimum of 10:1.

All study protocols were approved by the ethics committee of Nagasaki University Graduate School of Biomedical Sciences (approval no.: 23063001). The study was conducted in accordance with the ethical principles of the Declaration of Helsinki. Participation required the provision of informed consent, which was obtained from all respondents through the online platform prior to their involvement in the survey.

This study is reported in accordance with the STROBE (Strengthening the Reporting of Observational Studies in Epidemiology) guidelines for cross-sectional studies.

### 2.2 Data sources and measurement

Data were collected using a self-administered questionnaire delivered via an online survey platform. The survey was implemented in Japanese with identical wording, response options, and layout across regions to ensure comparability of measurements. The risk literacy scale was used in accordance with its published English and Japanese descriptions, and item content was consistent with the original instrument (19). Knowledge items were developed based on established scientific consensus on radiation risks and reviewed to ensure clarity and face validity. Mandatory responses were required for key items, thereby minimizing item non-response, and data were exported in anonymized form for analysis.

### 2.3 Variables

#### 2.3.1 Perceived heritable risk

The primary outcome was perceived heritable radiation risk, assessed using the following question: “To what extent do you think the current radiation exposure may affect future generations (children and grandchildren) of people in Fukushima Prefecture?”. Responses were recorded on a four-point Likert-type scale, from 1 (“very unlikely”) to 4 (“very likely”). For statistical analysis, these responses were dichotomized into “low perceived risk” (very unlikely or somewhat unlikely) and “high perceived risk” (somewhat likely or very likely).

#### 2.3.2 Radiation-related knowledge (independent variable)

Radiation knowledge was measured using five items covering key domains: (1) radiation units (Bq vs. Sv), (2) natural background radiation, (3) DNA repair mechanisms, (4) heritable effects among offspring of atomic bomb survivors, and (5) current scientific consensus on heritable effects following the Fukushima accident. Items 4 and 5 were adapted from previous research (12), while items 1–3 were developed based on expert opinions in radiation medical science to capture fundamental knowledge. For each item, participants selected “Yes,” “No,” or “Do not know.” Correct responses were coded as 1 and incorrect or “Do not know” responses as 0. A total knowledge score (range 0–5) was calculated, with higher scores indicating greater factual knowledge (exact item wording and rationale are provided in Supplementary Table S1). Given that the five items were designed to sample distinct content domains rather than to measure a single underlying knowledge trait, the total score is treated as an additive content-coverage index rather than a psychometrically validated unidimensional scale, consistent with common practice for factual knowledge indices in survey research.

#### 2.3.3 Risk literacy (independent variable)

Risk literacy was assessed using the 24-item scale developed by Kanazawa et al. (19), which has been validated for risk education research in Japan. The scale comprises six subscales: (F1) zero-risk orientation, (F2) trade-off between risk and benefit, (F3) trade-off between different risks, (F4) paradox of risk perception, (F5) basic knowledge of risk, and (F6) cognitive bias in risk perception. Items in Factors 1, 3, 4, and 6 were reverse-worded and were recoded prior to the CFA and all subsequent analyses. Each item was rated on a six-point Likert-type scale, from 1 (“strongly disagree”) to 6 (“strongly agree”). For each subscale, a mean score was calculated by averaging the four constituent items, with higher scores reflecting greater risk literacy in that domain. Although the original scale was developed and validated for subscale-level use, the present study additionally calculated an overall risk literacy score as the mean of all 24 items (range, 1–6) to provide a global index of risk literacy alongside the subscale-specific scores. Given the mixed positive/negative pattern of inter-factor correlations (Supplementary Table S3), this total score is treated as an exploratory summary measure of overall risk literacy rather than a validated unidimensional construct.

#### 2.3.4 Sociodemographic variables

Data on gender (male/female), age (categorized into five groups: 20s, 30s, 40s, 50s, and 60s, with the 20s group as the reference category), place of residence (Fukushima vs. Tokyo metropolitan area), and highest educational attainment (high school or below vs. university, junior college, or higher) were collected as covariates.

### 2.4 Statistical methods

Descriptive statistics were used to summarize participant characteristics, radiation-related knowledge, risk literacy, and concern about radiation effects by region. Continuous variables are presented as means and standard deviations (SDs), while categorical variables are shown as frequencies and percentages.

To confirm the factorial validity of the risk literacy scale, confirmatory factor analysis (CFA) was conducted using maximum likelihood estimation, with model fit evaluated using the comparative fit index (CFI), Tucker–Lewis Index (TLI), root mean square error of approximation (RMSEA), and standardized root mean square residual (SRMR). To evaluate whether the risk literacy scale could be validly compared across regions, measurement invariance was tested using multi-group confirmatory factor analysis (Fukushima vs. Kanto), following a configural–metric–scalar sequence. Given the sensitivity of the chi-square difference test to large sample sizes, invariance was additionally evaluated using the criterion of ΔCFI < 0.01 (20). Conventional cutoff values of CFI and TLI ≥ 0.90 and RMSEA and SRMR ≤ 0.08 were considered indicative of acceptable model fit (21). The internal consistency of each subscale was assessed using Cronbach’s alpha.

To explore factors associated with high concern about the heritable effects of radiation, bivariate analyses were performed. Chi-square tests were used for categorical variables, and the *t*-test was used to compare mean risk literacy scores between those with and without high concern. These bivariate comparisons were treated as descriptive and were not corrected for multiple testing, whereas Bonferroni correction (α = 0.0125) was applied to the four pre-specified subscale-specific mediation tests reported below, which were confirmatory in nature. Hierarchical logistic regression analysis was used to identify independent predictors of high concern about heritable effects. Variables were entered in four sequential steps: Model 1 included sociodemographic variables (gender, age group [20s as reference], educational attainment, and residential area); Model 2 additionally included radiation knowledge score; Model 3 additionally introduced the overall risk literacy score, retaining radiation knowledge, and Model 4 included knowledge and all six risk literacy subscales simultaneously. Age was categorized into five groups (20s, 30s, 40s, 50s, and 60s), consistent with the descriptive statistics, with the 20s group serving as the reference category. Although treating age as a continuous variable yielded comparable model fit (Δ in Akaike’s information criterion [AIC] < 1), a categorical approach was adopted to capture potential nonlinear associations. In the mediation analyses and the sensitivity analyses based on Model 4, age was entered as a continuous variable. Potential multicollinearity among the independent variables was assessed using the variance inflation factor (VIF), ensuring that all VIF values were < 5. Following the potential-outcomes framework for causal mediation analysis (22), we conducted an exploratory analysis decomposing the total association between radiation knowledge and concern into an average causal mediation effect (ACME) and an average direct effect (ADE), with risk literacy specified as the mediator, using quasi-Bayesian confidence intervals (CIs) with 1,000 simulations. Because the cross-sectional design precludes verification of the sequential ignorability assumption required for causal inference, the ACME and ADE are interpreted as a statistical decomposition of association consistent with a hypothesized mediating pathway, rather than as confirmed evidence of causal ordering. To assess robustness to violations of this assumption, a sensitivity analysis was conducted following Imai et al., examining the value of the sensitivity parameter ρ (the correlation between the error terms of the mediator and outcome models) at which the ACME would be reduced to zero. A random seed was set to ensure reproducibility of the simulation-based CIs.

$$\text{Proportion mediated}=\frac{\text{ACME}}{\text{ACME}+\mathrm{ADE}}$$

Several additional sensitivity analyses were conducted. A three-item knowledge score excluding two items whose content closely paralleled the outcome (heritable effects among atomic-bomb survivors’ offspring; scientific consensus on heritable effects following the Fukushima accident) was used to re-estimate the primary models and the mediation analysis. The mediation analysis was also repeated using the mean of the five risk literacy subscales excluding F6, given the content proximity between F6 and the outcome noted below. The dichotomized outcome was re-analyzed using proportional-odds ordinal logistic regression on the original four-point scale, with the proportional-odds assumption evaluated using the Brant test. Radiation knowledge was additionally entered as a categorical variable to evaluate the linearity assumption underlying its treatment as a continuous predictor. Region-stratified models and a model including residence × predictor interaction terms were estimated to formally test whether associations between cognitive factors and concern differed by region. Co-residence with children and grandchildren was examined as an additional covariate. Variance inflation factors (VIFs) were calculated for all predictors in the final model to assess multicollinearity.

All statistical analyses were performed using R software (version 4.4.2; R Foundation for Statistical Computing, Vienna, Austria) and RStudio interface (version 2024.12.1; Posit Software, Boston, MA), with a two-sided significance level of 0.05.

## 3 Results

A total of 1,529 responses were included in the final analysis. To verify the factorial structure of the risk literacy scale in the present sample, CFA was performed. The six-factor model yielded the following fit indices: χ^2^ (237) = 3082.47, CFI = 0.885, TLI = 0.866, RMSEA = 0.089 (90% CI: 0.086–0.091), SRMR = 0.094; all four indices fell outside the conventional thresholds (CFI/TLI ≥ 0.90; RMSEA/SRMR ≤ 0.08). Nonetheless, all standardized factor loadings were statistically significant (all *p* < 0.001), ranging from 0.57 to 0.91 (Supplementary Table S2), supporting the conceptual coherence of each subscale. All subscales demonstrated high internal consistency: F1 α = 0.903, F2 α = 0.833, F3 α = 0.833, F4 α = 0.879, F5 α = 0.867, and F6 α = 0.881. The full 24-item composite also demonstrated acceptable internal consistency (α = 0.80). Inter-factor correlations showed a mixed pattern of positive and negative associations among the subscales (Supplementary Table S3), consistent with their conceptual distinctiveness.

To evaluate whether the scale could be validly compared across regions, measurement invariance was tested using multi-group CFA (Fukushima vs. Kanto). Although the chi-square difference test indicated a significant decrement in fit when factor loadings were constrained (Δχ^2^ = 50.98, Δdf = 18, *p* < 0.001), this test is known to be oversensitive with large samples. Using the more robust ΔCFI criterion (< 0.01), both metric (ΔCFI = 0.001) and scalar (ΔCFI < 0.001) invariance were supported, indicating that regional comparisons of subscale scores are defensible.

Table 1 presents the demographic characteristics, radiation-related knowledge, risk literacy score, and concern about radiation effects of the study participants stratified by region (Fukushima Prefecture: *n* = 772 vs. Tokyo metropolitan area: *n* = 757). Significant regional differences were observed in age distribution (*p* = 0.002) and co-residence with children and grandchildren (*p* < 0.001). Regarding radiation-related knowledge (Q1–Q5), Fukushima residents demonstrated a significantly higher proportion of correct answers for Q2–Q5 and a lower proportion of “Do not know” responses across all items compared with Tokyo residents. For Q1, however, a supplementary two-by-two comparison indicated no significant regional difference in accuracy itself (11.9% vs. 10.3%, *p* = 0.357); the significant three-category result for this item primarily reflects differential ‘do not know’ response rates rather than differences in factual accuracy. Experience of radiation education did not differ by region. Concerns about the heritable effects of radiation were significantly higher in Tokyo (46.0%) compared with Fukushima (36.5%, *p* < 0.001). The total risk literacy score was significantly higher in Tokyo (mean, 3.55 ± 0.50 vs. 3.49 ± 0.50; *p* = 0.019, Cohen’s d = 0.12). Significant subscale differences favored Tokyo for F1 (zero-risk orientation) (3.52 ± 1.11 vs. 3.37 ± 1.13; *p* < 0.01, d = 0.14) and F5 (basic risk knowledge) (2.96 ± 1.01 vs. 2.86 ± 0.97; *p* < 0.05, d = 0.10), whereas a trend toward higher F6 (cognitive bias in risk perception) scores was observed for Fukushima (3.94 ± 0.99 vs. 3.85 ± 1.02; *p* = 0.081).

**Table 1:** Demographic factors, radiation risk perception, radiation knowledge accuracy, and risk literacy among residents in Fukushima and the Tokyo metropolitan area.

Variables	Units	Total (*N* = 1,529)	Fukushima (*n* = 772)	Tokyo (*n* = 757)	*p*-value
Gender	Male	777 (50.8%)	391 (50.6%)	386 (51.0%)	0.893
	Female	752 (49.2%)	381 (49.4%)	371 (49.0%)	
Age	20y	229 (15.0%)	106 (13.7%)	123 (16.2%)	0.002
	30y	278 (18.2%)	133 (17.2%)	145 (19.2%)	
	40y	353 (23.1%)	165 (21.4%)	188 (24.8%)	
	50y	331 (21.6%)	165 (21.4%)	166 (21.9%)	
	60y	338 (22.1%)	203 (26.3%)	135 (17.8%)	
Educational attainment	≤High school	547 (35.8%)	370 (47.9%)	177 (23.4%)	<0.001
	≥University	982 (64.2%)	402 (52.1%)	580 (76.6%)	
Household annual income (*n* = 1,214)	≤3,000,000 yen	345 (28.4%)	209 (34.7%)	136 (22.2%)	<0.001
	3,000,000 yen – 5,999,999 yen	420 (34.6%)	218 (36.2%)	202 (33.0%)	
	≥6,000,000 yen	449 (37.0%)	175 (29.1%)	274 (44.8%)	
Co-residence with children	Yes	722 (47.2%)	420 (54.4%)	302 (39.9%)	<0.001
	No	807 (52.8%)	352 (45.6%)	455 (60.1%)	
Co-residence with grandchildren	Yes	161 (10.5%)	115 (14.9%)	46 (6.1%)	<0.001
	No	1,368 (89.5%)	657 (85.1%)	711 (93.9%)	
Radiation education	Yes	674 (44.1%)	331 (42.9%)	343 (45.3%)	0.364
	No	855 (55.9%)	441 (57.1%)	414 (54.7%)	
Concerns about heritable effects of radiation	Yes	630 (41.2%)	282 (36.5%)	348 (46.0%)	<0.001
	No	899 (58.8%)	490 (63.5%)	409 (54.0%)	
Knowledge Q1: radiation units	Correct	170 (11.1%)	92 (11.9%)	78 (10.3%)	<0.001
	Incorrect	582 (38.1%)	345 (44.7%)	237 (31.3%)	
	Unknown	777 (50.8%)	335 (43.4%)	442 (58.4%)	
Knowledge Q2: background radiation	Correct	1,093 (71.5%)	586 (75.9%)	507 (67.0%)	<0.001
	Incorrect	79 (5.2%)	33 (4.3%)	46 (6.1%)	
	Unknown	357 (23.3%)	153 (19.8%)	204 (26.9%)	
Knowledge Q3: DNA repair	Correct	219 (14.3%)	125 (16.2%)	94 (12.4%)	0.011
	Incorrect	619 (40.5%)	325 (42.1%)	294 (38.8%)	
	Unknown	691 (45.2%)	322 (41.7%)	369 (48.7%)	
Knowledge Q4: A-bomb offspring effects	Correct	313 (20.5%)	185 (24.0%)	128 (16.9%)	0.003
	Incorrect	384 (25.1%)	189 (24.5%)	195 (25.8%)	
	Unknown	832 (54.4%)	398 (51.6%)	434 (57.3%)	
Knowledge Q5: Fukushima health effects	Correct	412 (26.9%)	240 (31.1%)	172 (22.7%)	<0.001
	Incorrect	308 (20.1%)	160 (20.7%)	148 (19.6%)	
	Unknown	809 (52.9%)	372 (48.2%)	437 (57.7%)	
Knowledge total score	Mean (SD)	1.44 (1.16)	1.59 (1.17)	1.29 (1.13)	<0.001
Risk literacy^a)^	Score (SD)	3.52 (0.50)	3.49 (0.50)	3.55 (0.50)	0.019
F1: Zero-risk orientation^a)^	Score (SD)	3.44 (1.12)	3.37 (1.13)	3.52 (1.10)	0.007
F2: Trade-off between risk and benefit^a)^	Score (SD)	3.47 (0.95)	3.43 (0.93)	3.52 (0.98)	0.064
F3: Trade-off between different risks^a)^	Score (SD)	3.97 (0.94)	3.97 (0.92)	3.98 (0.96)	0.879
F4: Paradox of risk perception^a)^	Score (SD)	3.45 (1.02)	3.40 (1.00)	3.50 (1.04)	0.064
F5: Basic knowledge of risk^a)^	Score (SD)	2.91 (0.99)	2.86 (0.97)	2.96 (1.01)	0.044
F6: Cognitive bias in risk perception^a)^	Score (SD)	3.90 (1.00)	3.94 (0.99)	3.85 (1.02)	0.081

a) Risk literacy dimensions are scored as mean values ranging from 1 to 6. Household annual income was reported by 1,214 of the 1,529 participants (79.4%); the remaining 315 participants (20.6%) selected “prefer not to answer” for this item.

Table 2 shows the results of a comparison of demographic characteristics, radiation knowledge, and risk literacy scores between residents with and without high concern about heritable effects. Heritable concerns were more frequently reported by females, residents of the Tokyo metropolitan area, and those with lower radiation-related knowledge scores (all *p* < 0.001). The total risk literacy score was significantly higher in the group without concerns (3.66 ± 0.49) than in the group with concerns (3.33 ± 0.46; *p* < 0.001, Cohen’s d = 0.69). Furthermore, participants without concerns scored significantly higher on subscales F1 (d = 0.48), F3 (d = 0.25), F4 (d = 0.54), and F6 (d = 0.67; all *p* < 0.001).

**Table 2:** Factors related to concerns about heritable effects from radiation exposure (chi-square analysis and *t*-test).

Variables	Units	No concern (*n* = 899)	Concern (*n* = 630)	*p*-value
Gender	Male	495 (63.7%)	282 (36.3%)	<0.001
	Female	404 (53.7%)	348 (46.3%)	
Age	20s	149 (65.1%)	80 (34.9%)	0.013
	30s	176 (63.3%)	102 (36.7%)	
	40s	196 (55.5%)	157 (44.5%)	
	50s	175 (52.9%)	156 (47.1%)	
	60s	203 (60.1%)	135 (39.9%)	
Residential area	Fukushima	490 (63.5%)	282 (36.5%)	<0.001
	Tokyo	409 (54.0%)	348 (46.0%)	
Educational attainment	≤High school	323 (59.1%)	224 (40.9%)	0.924
	≥University	576 (58.7%)	406 (41.3%)	
Household annual income (*n* = 1,214)	≤3,000,000 yen	188 (54.5%)	157 (45.5%)	0.042
	3,000,000 yen–5,999,999 yen	266 (63.3%)	154 (36.7%)	
	≥6,000,000 yen	272 (60.6%)	177 (39.4%)	
Co-residence with children	Yes	426 (47.4%)	296 (47.0%)	0.918
	No	473 (52.6%)	334 (53.0%)	
Co-residence with grandchildren	Yes	90 (10.0%)	71 (11.3%)	0.481
	No	809 (90.0%)	559 (88.7%)	
Radiation education	Yes	411 (61.0%)	263 (39.0%)	0.137
	No	488 (57.1%)	367 (42.9%)	
Radiation knowledge (range 0–5)	Score (SD)	1.64 (1.26)	1.17 (0.94)	<0.001
Risk literacy	Score (SD)	3.66 (0.49)	3.33 (0.46)	<0.001
F1: Zero-risk orientation	Score (SD)	3.66 (1.13)	3.14 (1.04)	<0.001
F2: Trade-off between risk and benefit	Score (SD)	3.49 (0.97)	3.45 (0.93)	0.400
F3: Trade-off between different risks	Score (SD)	4.07 (0.90)	3.83 (0.98)	<0.001
F4: Paradox of risk perception	Score (SD)	3.67 (0.99)	3.14 (0.99)	<0.001
F5: Basic knowledge of risk	Score (SD)	2.90 (0.96)	2.91 (1.04)	0.909
F6: Cognitive bias in risk perception	Score (SD)	4.16 (0.95)	3.52 (0.97)	<0.001

*p*-values were calculated using the chi-square test for categorical variables and the independent t test for continuous variables. Risk literacy dimensions are scored as mean values ranging from 1 to 6.

The proportion of respondents with high concern showed a graded association with increasing radiation knowledge, declining overall from 43.5% at a score of 0 to 5.3% at a score of 5, although the association was not strictly monotonic (concern was highest, at 52.9%, among respondents scoring 1; Figure 1A). A sensitivity analysis entering knowledge as a categorical rather than continuous predictor confirmed this pattern (Supplementary Table S4). The proportion of respondents with high concern also declined monotonically across risk literacy quartiles, from 60.6% in the lowest quartile to 20.1% in the highest (Figure 1B).

**Figure 1:**
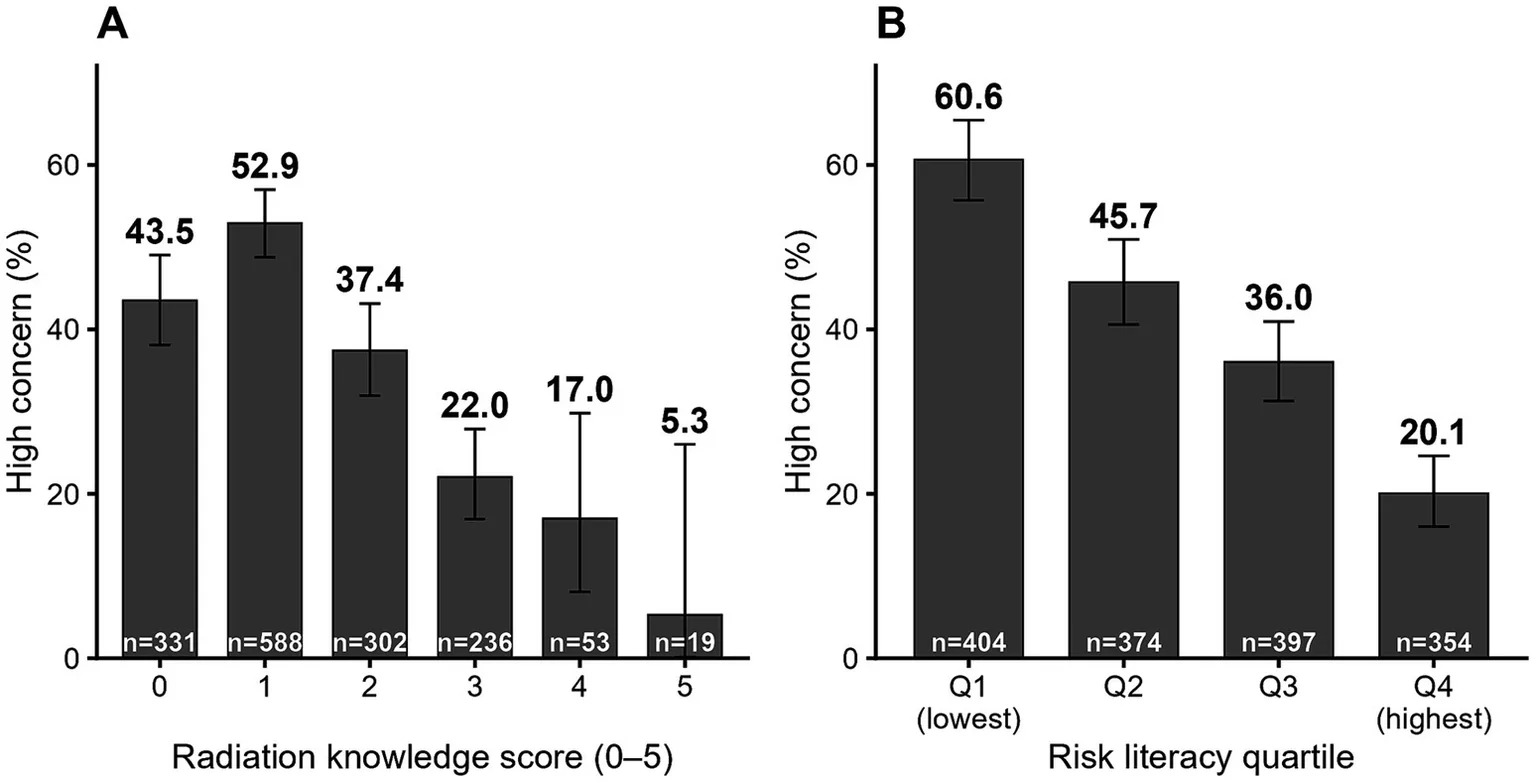
Graded associations between cognitive factors and concern about the heritable effects of radiation. **(A)** Proportion with high concern by radiation knowledge score (0–5); numbers of respondents at each level: *n* = 331, 588, 302, 236, 53, and 19. **(B)** Proportion with high concern by risk literacy quartile (Q1 = 1.54–3.25, Q2 = 3.25–3.50, Q3 = 3.50–3.79, Q4 = 3.79–5.54); *n* = 404, 374, 397, and 354. Error bars represent 95% confidence intervals for the proportion in each category. Both associations were statistically significant (χ^2^ test, both *p* < 0.001).

Table 3 presents the results of the hierarchical multivariable logistic regression analysis. Model 1 included only the control variables (gender, age, education, and residence), and revealed that the residence location was a significant predictor (OR = 1.53, 95% CI [1.23, 1.89], *p* < 0.001). In Model 2, the addition of radiation knowledge significantly improved model fit (Δχ^2^ = 50.07, Δdf = 1, *p* < 0.001, AIC = 1996.2), and was itself significantly associated with reduced concern (OR = 0.71, *p* < 0.001). In Model 3, the overall risk literacy score was introduced, resulting in a further significant improvement in fit (Δχ^2^ = 138.51, Δdf = 1, *p* < 0.001, AIC = 1858.6). In the final step (Model 4), the overall score was replaced by the six subscales of risk literacy, which yielded the lowest AIC (1837.8) among all models.

**Table 3:** Hierarchical multiple logistic regression analysis predicting concerns about heritable effects.

Variables	Model 1	Model 2	Model 3	Model 4
Gender (ref: male)	1.52 (1.24–1.87)^***^	1.34 (1.09–1.69)^**^	1.06 (0.84–1.32)	1.07 (0.85–1.35)
Age (ref: 20s)	-	-	-	-
30s	1.09 (0.76–1.58)	1.07 (0.74–1.56)	0.98 (0.66–1.46)	1.04 (0.70–1.56)
40s	1.51 (1.07–2.14)^*^	1.51 (1.06–2.14)^*^	1.30 (0.90–1.89)	1.41 (0.97–2.07)
50s	1.70 (1.20–2.42)^**^	1.76 (1.24–2.52)^**^	1.41 (0.97–2.06)	1.54 (1.05–2.27)^*^
60s	1.31 (0.92–1.86)	1.41 (0.99–2.03)	1.09 (0.71–1.52)	1.15 (0.78–1.71)
Educational attainment (ref: ≤high school)	0.93 (0.74–1.16)	1.05 (0.83–1.31)	1.21 (0.95–1.54)	1.19 (0.93–1.52)
Residential area (ref: Fukushima)	1.53 (1.23–1.89)^***^	1.36 (1.09–1.69)^**^	1.49 (1.18–1.87)^**^	1.42 (1.13–1.80)^**^
Radiation knowledge (score 0–5)	-	0.71 (0.64–0.78)^***^	0.75 (0.68–0.83)^***^	0.77 (0.69–0.85)^***^
Risk literacy	-	-	0.22 (0.17–0.29)^***^	-
F1: Zero-risk orientation ^a)^	-	-	-	0.94 (0.82–1.08)
F2: Trade-off & benefits ^a)^	-	-	-	0.79 (0.68–0.92)^**^
F3: Trade-off & risks ^a)^	-	-	-	1.04 (0.88–1.22)
F4: Cognitive paradox ^a)^	-	-	-	0.71 (0.60–0.83)^***^
F5: Basic knowledge of risk ^a)^	-	-	-	0.79 (0.69–0.91)^**^
F6: Risk perception biases ^a)^	-	-	-	0.54 (0.45–0.65)^***^
Goodness-of-fit				
AIC	2044.5	1996.2	1858.6	1837.8

*N* = 1,529. Values represent Odds Ratios (OR) with 95% confidence intervals in parentheses. a) Risk literacy dimensions are scored as mean values ranging from 1 to 6. Variance inflation factors for Model 4 ranged from 1.06 (age) to 2.04 (F6), well below the conventional threshold of 5. b) The Model 3 risk literacy score (24-item composite, SD = 0.50) is not directly comparable in magnitude to the Model 4 subscale scores (SDs, 0.95–1.13). *p < 0.05, **p < 0.01, *** *p* < 0.001.

In this final model, radiation knowledge remained a significant predictor of reduced concern (OR = 0.77, 95% CI [0.69, 0.85], *p* < 0.001). Among the risk literacy dimensions, four subscales demonstrated significant independent associations with lower concern: trade-offs and benefits (F2: OR = 0.79, *p* < 0.01), cognitive paradox (F4: OR = 0.71, *p* < 0.001), basic knowledge (F5: OR = 0.79, *p* < 0.001), and risk perception biases (F6: OR = 0.54, *p* < 0.001), with F6 showing the strongest association. Variance inflation factors for all predictors in the final model were below the conventional threshold of 5 (Table 3). To express these associations in absolute terms, marginal standardization (g-computation) estimated predicted probabilities of high concern at different predictor levels, holding other covariates at their observed values. Setting radiation knowledge to 0 versus 5 raised the predicted probability of high concern by 25.9 percentage points (prevalence ratio = 2.13). For F6, given the small number of respondents near the scale minimum and maximum (*n* = 34 and 117, respectively), the interquartile range was used instead: predicted probability was 1.52 times higher at the 25th versus 75th percentile.

To formally evaluate whether these associations differed by region, a model including residence × predictor interaction terms for radiation knowledge and all six risk literacy subscales was estimated. None of the interaction terms reached statistical significance (all *p* > 0.13; Supplementary Table S5), indicating that no regional difference in these associations was detected; given the sample size, this finding should be interpreted as an absence of detected interaction rather than confirmed evidence of uniformity across regions. Region-stratified estimates are reported in Supplementary Table S5.

Several sensitivity analyses supported the robustness of the primary findings. Household annual income, although associated with concern in bivariate analysis, was not significant when added to Model 4 in the subsample with complete income data (*n* = 1,214; OR = 0.96, 95% CI [0.82, 1.13]), and did not materially alter the other coefficients. Re-estimating Model 4 with the original four-point outcome using proportional-odds ordinal logistic regression yielded a highly consistent pattern of associations (e.g., knowledge: OR = 0.76, 95% CI [0.70, 0.83]; F6: OR = 0.48, 95% CI [0.41, 0.56], both *p* < 0.001), and the proportional-odds assumption was supported (Brant test, omnibus χ^2^ = 25.86, *df* = 22, *p* = 0.26). Adding co-residence with children and grandchildren as covariates did not meaningfully change the pattern of associations (e.g., knowledge: OR = 0.76; F6: OR = 0.54), and neither covariate was independently significant (children: OR = 1.14, *p* = 0.30; grandchildren: OR = 1.04, *p* = 0.82). Institutional trust and attitudes toward expert-led decision-making, when added to Model 4, were each significantly associated with concern (institutional trust: OR = 0.57, 95% CI [0.46, 0.71]; expert-led decision-making: OR = 1.25, 95% CI [1.04, 1.50]), and the residence coefficient was materially unchanged (OR = 1.45 without vs. 1.48 with these variables; age entered as a continuous variable, cf. OR = 1.42 with categorized age in Table 3).

By contrast, when radiation knowledge was restricted to three items assessing general radiological and biological principles—excluding two items whose content closely paralleled the outcome (heritable effects among atomic-bomb survivors’ offspring; scientific consensus on heritable effects following the Fukushima accident)—its association with concern was no longer statistically significant (OR = 0.97, 95% CI [0.82, 1.16]). Repeating the mediation analysis with this three-item index yielded ACME = −0.028 (95% CI [−0.041, −0.014], *p* < 0.001) and ADE = −0.011 (95% CI [−0.047, 0.024], *p* = 0.548), with a total effect of −0.039 (95% CI [−0.076, −0.003], *p* = 0.042; approximately 70% mediated); the direct effect was no longer statistically significant. This significant total effect is consistent with the non-significant estimate in the primary model: the latter is conditional on the six risk literacy subscales and thus corresponds more closely to the direct effect (*p* = 0.548), whereas the total effect, adjusted for sociodemographic variables only, also includes the association operating through risk literacy.

An exploratory statistical mediation analysis, using quasi-Bayesian confidence intervals (1,000 simulations), examined the total association between radiation knowledge and heritable concerns (total effect = −0.091, 95% CI [−0.114, −0.067], *p* < 0.001) decomposing it into an average causal mediation effect (ACME = −0.027, 95% CI [−0.035, −0.018], *p* < 0.001) and an average direct effect (ADE = −0.064, 95% CI [−0.087, −0.041], *p* < 0.001). The proportion of the total effect mediated through risk literacy was 29.2% (95% CI [20.1%, 42.2%]) (Figure 2). Because the risk literacy composite used as the mediator has a compressed variance relative to its constituent subscales (see Table 3, note), the magnitude of the indirect effect should be interpreted with this scaling in mind rather than compared directly to subscale-level associations. A sensitivity analysis indicated that an unmeasured confounder would need to induce a correlation of ρ = −0.3 between the error terms of the mediator and outcome models to reduce the ACME to zero.

**Figure 2:**
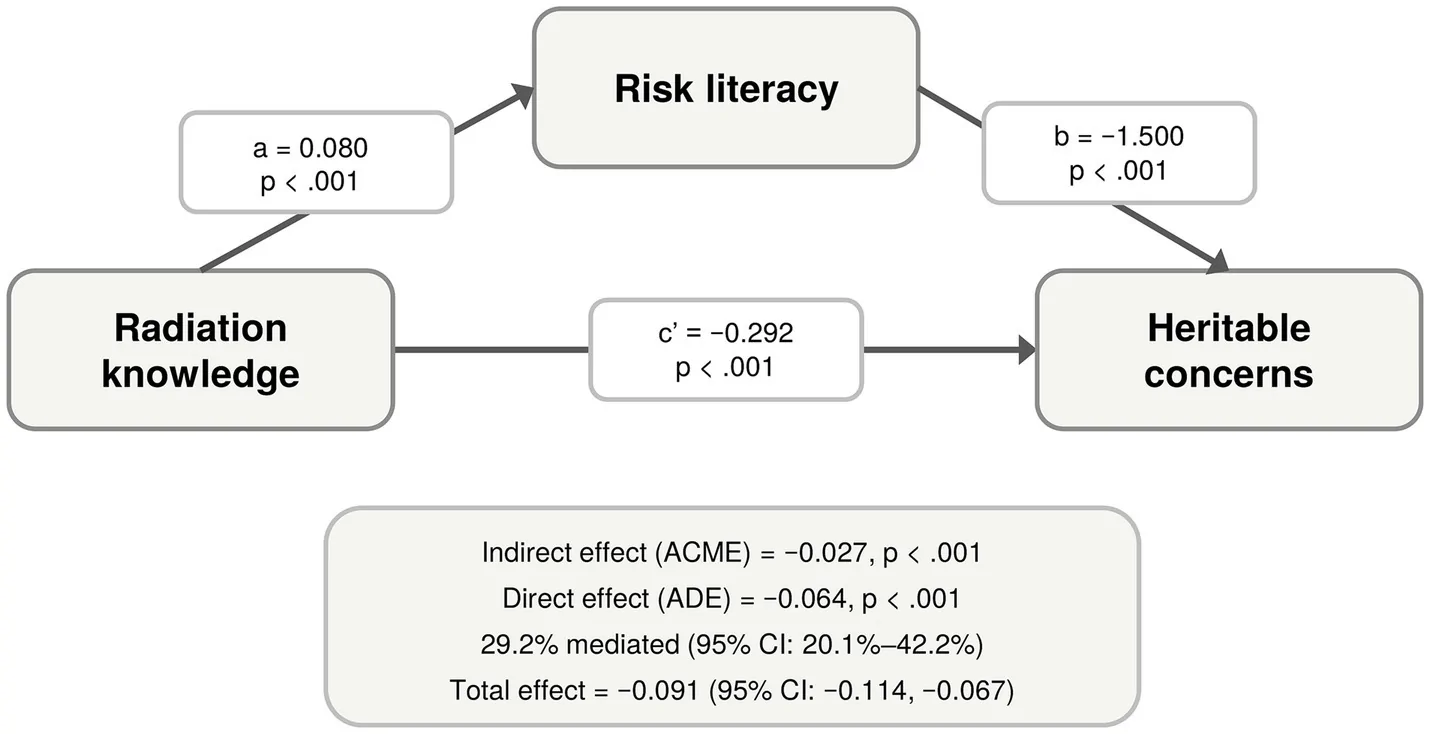
The mediating effect of risk literacy. Path diagram of the exploratory mediation analysis. Path a is the unstandardized coefficient from linear regression; paths b and c’ are unstandardized log-odds coefficients from logistic regression. The summary box reports the average causal mediation effect (ACME), average direct effect (ADE), proportion mediated, and total effect, expressed as average effects on the outcome probability scale. All models were adjusted for gender, age, residential area, and educational attainment. Confidence intervals estimated using quasi-Bayesian simulation (1,000 iterations). ACME, average causal mediation effect; ADE, average direct effect; CI, confidence interval.

To identify which risk literacy dimensions accounted for this mediation effect, exploratory subscale-specific analyses were conducted for the four subscales significantly associated with concern in the multivariable model (F2, F4, F5, and F6). After Bonferroni correction (α = 0.0125), only F6 (cognitive bias in risk perception) showed a significant indirect effect (ACME = −0.023, 95% CI [−0.031, −0.015], proportion mediated = 25.9%), whereas F2, F4, and F5 did not (Supplementary Table S6; Supplementary Figure S1). However, when the five subscales excluding F6 were combined into a single composite mediator, a significant indirect association remained (21.7% mediated, 95% CI [13.9%, 32.7%]), indicating that the overall indirect association was not attributable to F6 alone.

## 4 Discussion

This study examined the relationship between radiation knowledge, risk literacy, and concerns about the heritable effects of radiation among residents of Fukushima Prefecture and the Tokyo metropolitan area. A key finding of this study was the distinct geographical variation in radiation-related knowledge and heritable concerns between the two regions. While residents of Fukushima demonstrated significantly higher knowledge accuracy regarding radiation than those in the Tokyo metropolitan area, their level of concern about heritable effects was significantly lower. This finding directly aligns with previous research demonstrating that the geographical gap in radiation risk perception after the Fukushima accident is substantially mediated by the factual knowledge acquired by individuals (12). Through localized risk communication, community briefings, and continuous public relations by local governments over the decade following the 2011 accident, Fukushima residents may have had substantial opportunities to update their scientific evidence regarding radiation (5, 6, 23), including international consensus reports indicating no discernible increase in heritable effects in post-accident populations (2). However, this account should be interpreted cautiously: the proportion of respondents reporting prior radiation education did not differ significantly between regions (42.9% vs. 45.3%, *p* = 0.364; Table 1), which runs counter to a straightforward risk-communication explanation. By contrast, for residents of Tokyo, radiation issues have largely faded from daily discourse, limiting opportunities to update radiation knowledge. Without sufficient domain-specific updates, individuals outside the directly affected region may be more prone to overestimating unfamiliar or emotionally salient risks connected to invisible hazards (24). This pattern is consistent with the risk perception paradox, whereby risk perception is shaped less by objective knowledge than by experiential, contextual, and trust-related factors (14). Consequently, these findings may reflect unequal long-term exposure to risk information as one contributing factor to the gap in concern, reinforcing the need for sustained nationwide risk education. It should also be noted that the outcome item itself may not be interpretively neutral across regions. Because the item asks respondents to judge effects on future generations “of people in Fukushima Prefecture,” it constitutes a self-referent judgment for Fukushima respondents but an other-referent judgment for Kanto respondents—an asymmetry that is arguably inherent to any study of this accident, given that the affected population is, by definition, geographically situated. Although self-other asymmetries in risk judgment are well documented (25, 26), their interpretation as a genuine psychological bias has since been questioned (27, 28). Nonetheless, this inherent framing asymmetry cannot be ruled out as a contributing factor to the observed regional gap, independent of the cognitive factors examined here.

The results of the multivariable logistic regression analysis further indicated that higher radiation-related knowledge was independently associated with decreased heritable concerns. This five-item knowledge scale evaluated participants’ understanding of both fundamental physical concepts and epidemiological facts. The results indicated that possessing accurate scientific facts may serve as a critical cognitive foundation, enabling individuals to evaluate risk rationally and reducing the amplified dread or subjective fear often tied to ionizing radiation (24). This finding is consistent with previous studies within Fukushima Prefecture, which demonstrated that a correct understanding of specific biological mechanisms, such as cellular DNA repair and the absence of confirmed heritable effects, correlates with reduced next-generation anxiety among residents (18). This educational mitigation effect is further supported by evidence from diverse cohorts, including medical health sciences students (29) and certified disaster prevention advisers (30). Notably, while bivariate analyses suggested a nonlinear pattern in which concern peaked among middle-aged groups—particularly those in their 50s, who were of childbearing age or had dependent children at the time of the accident—this age effect was substantially attenuated after adjustment for knowledge and risk literacy. Although the 50s age group retained a modest independent association in the full model, the marked reduction in the age coefficients indicated that age-related variation in concern is largely, though not entirely, accounted for by the underlying acquisition of factual knowledge and structured risk literacy. By identifying an independent association between higher knowledge scores and decreased heritable concerns in this population, the present findings support the value of fact-based education as a key component of efforts to mitigate unwarranted public anxiety. However, a sensitivity analysis restricting the knowledge score to three items assessing general radiological and biological principles—excluding two items whose content closely paralleled the outcome (heritable effects among atomic-bomb survivors’ offspring; scientific consensus on heritable effects following the Fukushima accident)—rendered this association non-significant (OR = 0.97, 95% CI [0.82, 1.16]). This indicates that, analogous to the F6 finding discussed below, part of the apparent inverse association between knowledge and concern may reflect content overlap with the outcome—in effect, asking respondents whether they already know the answer to the same empirical question the outcome measure asks them to judge—rather than a broader association with factual knowledge. This finding tempers claims regarding the generalizability of knowledge as a protective factor. In addition, the association between knowledge and concern was not strictly monotonic, with concern highest among respondents scoring 1 rather than 0 before declining progressively (Figure 1A)—a pattern consistent with evidence from other risk domains that information provision does not always reduce risk perception (31). This may partly reflect the coding of “do not know” responses as incorrect and the sparse uppermost categories (*n* = 53 and 19 at scores 4 and 5), warranting caution in interpreting the higher end of the scale.

This exploratory mediation analysis further examined the statistical structure of this association. Approximately 29.2% of the total association between radiation knowledge and heritable concerns was statistically mediated by risk literacy, while the remaining 70.8% was independent of risk literacy (Figure 2). For the full five-item knowledge score, this pattern suggests that factual knowledge is associated with lower concern predominantly through pathways other than the risk-literacy subscales measured here—for example, by directly resolving specific misconceptions about the heritability of radiation effects—with an additional, smaller portion of the association operating through risk literacy. This pattern was, however, approximately reversed when the knowledge score was restricted to the three items on general radiological and biological principles: approximately 70% of the total association was then statistically mediated by risk literacy, and the direct effect was no longer significant. Read together, the two analyses suggest that the direct association observed for the full score is carried largely by the two items on heritable effects that parallel the outcome, and that the association of general radiation knowledge with concern overlaps with risk literacy more than the full-score decomposition implies, although this proportion is imprecise because the total effect for general knowledge was small, whereas the indirect association itself was clearly significant. A sensitivity analysis indicated that an unmeasured confounder would need to induce a correlation of ρ = −0.3 between the error terms of the mediator and outcome models to reduce this indirect effect to zero, suggesting moderate robustness to violations of the sequential ignorability assumption underlying this analysis. However, exploratory subscale-specific analyses indicated that this indirect pathway was almost entirely attributable to a single dimension—F6 (cognitive bias in risk perception)—which alone accounted for a proportion mediated of 25.9%, whereas the indirect effects through F2, F4, and F5 were negligible and non-significant after correction (Supplementary Table S6, Supplementary Figure S1). When the five subscales excluding F6 were instead combined into a single composite mediator, a significant indirect effect remained (21.7% mediated, 95% CI [13.9%, 32.7%]), indicating that the overall mediation was not attributable to F6 alone. In particular, the F6 items concern radiation- and dose–response-specific beliefs, such as the conviction that any minute dose inevitably causes severe harm, or that artificial radiation is inherently more harmful than an equivalent dose of natural radiation. These items are closely related to the outcome domain, and the perceived asymmetry between artificial and natural hazards reflects a well-documented affect-driven bias, whereby judgments of risk are shaped by intuitive emotional responses rather than analytical evaluation (32, 33). Consequently, the mediation observed through F6 may be more parsimoniously interpreted as knowledge correcting radiation-specific misconceptions that are structurally close to the outcome, rather than as evidence that factual knowledge fosters broad, domain-general risk literacy. At the same time, because all variables were measured concurrently, the sequential ignorability assumption required for causal interpretation cannot be verified; reverse pathways—for example, lower baseline concern leading to greater knowledge acquisition—remain equally plausible. Given the cross-sectional design and this content proximity, risk literacy and knowledge are perhaps better understood as largely independent, complementary predictors of concern, rather than as a strictly sequential pathway.

By decomposing risk literacy into structured subscales rather than relying solely on a total aggregate score, this study characterized the nuanced cognitive mechanisms underlying public risk perception. Notably, the subscales showed a mixed pattern of positive and negative inter-factor correlations (Supplementary Table S3); for instance, F1 (zero-risk orientation) and F2 (trade-off acceptance) were negatively correlated. Although prior validation of this scale has not reported such a pattern, one possible interpretation is that these two subscales reflect conceptually opposing orientations toward risk—rejecting any level of risk versus actively weighing risks against benefits—which may manifest as a negative association even after reverse-scoring. In the final multivariable model (Table 3, Model 4), the initial geographical effect of residence location was somewhat attenuated but persisted (OR = 1.42, *p* = 0.003, compared with OR = 1.53, *p* < 0.001 in Model 1), after adjusting for these risk literacy subscales. Beyond the predictive value of Factor 6 (Risk perception bias, OR = 0.54, *p* < 0.001), several dimensions of risk literacy—including Factor 2 (Trade-off between risk and benefit, OR = 0.79), Factor 4 (Paradox of risk perception, OR = 0.71), and Factor 5 (Basic knowledge on risk, OR = 0.79)—were concurrently and significantly associated with lower concern. In accordance with the conceptual framework established by Kanazawa et al. (19), these dimensions collectively evaluate an individual’s capacity for systematic risk reasoning and structural understanding, such as recognizing that risk cannot be reduced to absolute zero and that decision-making inherently involves trade-offs. Importantly, bivariate comparisons (Table 1) indicated that the baseline levels of these risk literacy subscales did not fundamentally differ between the two regions, with Tokyo residents even demonstrating slightly higher scores in certain dimensions. This indicates that a general orientation or a baseline capacity for risk literacy is less influential than the active application of systematic, trade-off-based reasoning and awareness of cognitive biases when managing specific anxieties regarding heritable effects. Subscales F2, F4, and F5 employ generic, non-radiation-specific content (e.g., science, technology, food additives, pesticides), consistent with the original conceptualization of risk literacy as a domain-general construct (19). Their independent association with lower concern therefore reinforces the value of these dimensions as transferable cognitive resources, applicable beyond the immediate context of radiation. The F5 items assess self-reported confidence in probabilistic reasoning (e.g., the ability to intuitively grasp low-probability events) rather than objective factual knowledge, and may therefore reflect self-efficacy in risk reasoning rather than objective risk knowledge per se. Correlations between the objective knowledge score and all six risk literacy subscales were uniformly weak (r = 0.06–0.21), consistent with knowledge and risk literacy capturing largely distinct constructs. It should also be noted that, given the substantial correlations among several subscales (Supplementary Table S3), the Model 4 coefficients are mutually adjusted rather than fully independent, and should be interpreted as conditional on the other subscales rather than as evidence that each operates in isolation. A formal test of whether the associations for radiation knowledge and the six risk literacy subscales differed by region (residence × predictor interaction terms) did not reach significance for any predictor (all *p* > 0.13), indicating that no regional difference in these associations was detected. Given the sample size, this should be interpreted as an absence of detected interaction rather than confirmed evidence of uniformity across regions.

These findings have important implications for risk communication and public health policy. Domain-specific knowledge and multi-dimensional risk literacy appear to function as independent, complementary determinants of concern, each contributing an association of comparable magnitude. This complementary relationship parallels contemporary health literacy frameworks, which emphasize that factual knowledge and higher-order reasoning skills must work together to enable individuals to make autonomous health decisions (15, 16). While domain-specific knowledge serves as a necessary basis for addressing immediate radiological misconceptions, general risk-reasoning competencies—particularly the capacity to navigate trade-offs and recognize cognitive biases—may function as transferable protective factors that extend beyond the specific context of radiation. Even within remote cohorts subject to information fading, where emotional consequences and subjective fears regarding nuclear disasters are historically prone to persist over the long term (34), individuals equipped with robust structural reasoning may be better able to regulate their emotional reactions. Consequently, long-term public health strategies cannot rely solely on the one-way transmission of scientific facts. Just as the field has shifted from information-deficit approaches toward dialogue and trust-building (35, 36), our findings point to a complementary strategy: cultivating the audience’s own capacity for structured risk reasoning, enabling individuals to interpret risk information regardless of its source. To build resilience against persistent stigma and unwarranted dread, risk education should therefore address both dimensions in parallel rather than treating factual instruction and risk-reasoning skill-building as interchangeable. Prior experience in Fukushima Prefecture, where structured health literacy training was adapted and implemented for local public health nurses serving as community contact points (37), demonstrated the feasibility of capacity-building interventions in directly affected settings. However, the applicability of such models to non-affected regions is not straightforward, as the Fukushima approach was predicated on residents’ status as directly affected stakeholders and on the presence of public health nurses as established community intermediaries—conditions largely absent in metropolitan areas such as Tokyo, where residents are non-stakeholders with limited intrinsic interest (8). Given that no regional difference in the association between cognitive factors and concern was detected, risk education for non-affected populations may nonetheless benefit from embedding radiation-specific content within broader, transferable risk literacy curricula, delivered through general education channels rather than through the stakeholder-oriented, intermediary-based models effective in affected communities.

This study has several limitations that should be considered when interpreting the findings. First, the generalizability of the results is constrained by the inherent characteristics of online survey panels. To optimize population representativeness, the sampling was pre-stratified and allocated to match the actual demographic distributions of the target prefectures. Furthermore, using an incentivized online panel offered a distinct methodological advantage: it effectively captured responses from individuals who lacked inherent interest in radiation issues, thereby mitigating the self-selection bias often found in traditional mail-in surveys where only highly motivated or anxious individuals respond. The provider’s standard data-cleaning protocol, applied prior to data delivery, further excluded respondents exhibiting excessively short completion times or straight-lining on scale items. Nevertheless, because the panel relies on non-probabilistic sampling and excludes individuals without Internet access—particularly children and older adults—potential selection bias cannot be entirely eliminated. Second, reliance on self-reported data means that social desirability and recall biases cannot be excluded, particularly regarding subjective concerns and prior radiation education, and may operate asymmetrically between regions given the self-referent versus other-referent framing of the outcome item discussed above, since social desirability may be more salient when respondents evaluate their own concern than when evaluating concern about others. Third, the cross-sectional design precludes causal inference, and residual confounding due to unmeasured factors, such as media exposure, may have influenced the results. Institutional trust and attitudes toward expert-led decision-making were measured but not incorporated into the primary analysis, as these constructs fell outside the theoretical scope of the present study; their relationship with radiation-related concern is being examined separately. A sensitivity analysis incorporating both variables left the regional association materially unchanged (OR = 1.45 without vs. 1.48 with these variables). A comparison of the Ibaraki subsample (*n* = 36), which hosts nuclear facilities, with the remaining Kanto prefectures detected no difference in knowledge or concern, though the small subsample limits the power of this comparison. The exploratory mediation analysis, in particular, rests on the sequential ignorability assumption, which cannot be verified with the present data. The six-factor risk literacy structure also showed suboptimal fit in the confirmatory factor analysis, although factor loadings were significant and measurement invariance across regions was supported. In addition, the mediating role of risk literacy warrants cautious interpretation. The overall indirect effect was driven substantially by the F6 subscale, whose items overlap substantially with radiation- and dose–response-specific beliefs closely related to the outcome domain; this content proximity may have inflated the apparent mediation relative to the more domain-general subscales, although a significant indirect association remained even when F6 was excluded. A parallel concern applies to the knowledge score itself: two of its five items closely paralleled the outcome construct, and excluding them rendered the knowledge–concern association non-significant in the primary model. Furthermore, the subscale-specific mediation analyses used individual subscales as single mediators rather than a simultaneous multiple-mediator model, and should therefore be regarded as exploratory. The primary outcome was also dichotomized from an original four-point scale; although a sensitivity analysis using ordinal logistic regression yielded a highly consistent pattern of associations, this entails some loss of information. Finally, a conceptual limitation lies in the measurement of risk literacy. Although we utilized a structured, multidimensional risk literacy scale (19), empirical accumulation validating its psychometric stability and international generalizability across diverse hazard contexts remains relatively limited. This normative framing—that concern exceeding the evidence for detectable risk may be considered unwarranted—reflects a particular stance within risk communication ethics that not all readers may share. Despite these limitations, the large sample size and consistent associations across analyses support the internal validity of the findings, offering a scalable approach to monitor public risk perceptions in post-disaster contexts. Because the outcome prevalence was relatively high (41.2%), odds ratios may overstate the magnitude of association relative to risk ratios in this sample. Marginal standardization indicated that the prevalence ratio for radiation knowledge (comparing scores of 0 vs. 5) was 2.13 (risk difference = 25.9 percentage points), and for F6 (highest vs. lowest quartile) was 1.52, providing a complementary metric for interpreting the magnitude of these associations.

## 5 Conclusion

In conclusion, both radiation knowledge and multidimensional risk literacy were independently associated with concerns about the heritable effects of radiation—although the knowledge association was attenuated when items closely resembling the outcome were excluded, indicating some content overlap—and the geographical disparity in concern between Fukushima and Tokyo residents was statistically associated with these cognitive factors, although a significant residence effect remained after adjustment (OR = 1.42, *p* = 0.003), indicating that regional differences were only partly explained by knowledge and risk literacy. Although part of the association between knowledge and concern was statistically mediated by risk literacy, this mediation was concentrated in a single, radiation-specific dimension, suggesting that knowledge and general risk-reasoning competencies operate largely as complementary, independent determinants rather than as a simple sequential pathway. Residents outside the affected area demonstrated lower knowledge and greater concern, underscoring the importance of extending sustained risk communication nationwide. These findings suggest that risk education should not treat factual instruction and risk-reasoning skill-building as interchangeable, but rather, should cultivate both in parallel—correcting domain-specific misconceptions while fostering transferable competencies in trade-off reasoning and cognitive bias awareness—to promote informed decision-making and build resilient communities in post-nuclear contexts.

## Data Availability

The raw data supporting the conclusions of this article will be made available by the authors, without undue reservation.
