# Supplementary material for: Radiation knowledge, risk literacy, and concern about heritable effects: a cross-sectional study in Fukushima and the Tokyo metropolitan area
Source: Front Public Health. 2026 Oct 14;14:1977741. doi: 10.3389/fpubh.2026.1977741 (PMC13713507; doi:10.3389/fpubh.2026.1977741)
Supplement: Supplementary file 1 [file Supplementary_file_1.DOCX]

Supplementary Material

# 1 Supplementary Figures and Tables

**Supplementary Table S1.** Wording, correct answers, and rationale for the five radiation knowledge items

| **#** | **Item wording (English)** | **Correct answer** | **Rationale** |
| --- | --- | --- | --- |
| 1 | The unit representing radioactivity is the sievert (Sv), and the unit representing radiation's effect on people is the becquerel (Bq). | No | This statement reverses the correct definitions: the becquerel (Bq) measures radioactivity (the rate of radioactive decay of a source), whereas the sievert (Sv) measures the biological/health effect (effective dose) to humans. |
| 2 | Radiation exists in nature, and everyone receives a certain amount of exposure from natural background radiation. | Yes | Natural background radiation (from cosmic rays, terrestrial sources, and internal sources such as potassium-40) is ubiquitous, and all individuals are continuously exposed to it. |
| 3 | DNA (the physical basis of genes) damaged by radiation cannot be repaired. | No | Cells possess multiple DNA repair mechanisms (e.g., base excision repair, nucleotide excision repair, double-strand break repair) that correct much radiation-induced DNA damage; damage is not uniformly irreparable. |
| 4 | Large-scale studies, including of the offspring of atomic-bomb survivors, have found no increase in health effects among subsequent generations attributable to parental radiation exposure. | Yes | Long-term epidemiological studies of atomic-bomb survivors' offspring (e.g., Radiation Effects Research Foundation studies) have found no statistically significant increase in heritable effects attributable to parental radiation exposure. |
| 5 | It is presumed that radiation exposure from the Fukushima nuclear accident will not cause an increase in health effects among subsequent generations. | Yes | Given the substantially lower radiation doses associated with the Fukushima Daiichi accident compared with the atomic bombings, current scientific consensus (e.g., UNSCEAR) does not anticipate a detectable increase in heritable effects among descendants of exposed individuals. |

**Supplementary Table S2.** Risk literacy scale items and standardized factor loadings

| Factor | Item | Item content (author's English translation) | Standardized loading | SE |
| --- | --- | --- | --- | --- |
| F1: Zero-risk orientation | 1 | Highly dangerous technologies should not be used, no matter how convenient or useful they are. | 0.809 | 0.010 |
|  | 2 | Any technology with even a slight risk should not be used. | 0.901 | 0.007 |
|  | 3 | All chemical substances harmful to the human body should be banned. | 0.831 | 0.009 |
|  | 4 | Even if the probability of an accident is very low, technologies that could cause mass casualties at once should not be used. | 0.821 | 0.010 |
| F2: Trade-off between risk and benefit | 5 | In judging whether a technology is good or bad, it is acceptable to prioritize convenience over risk. | 0.735 | 0.015 |
|  | 6 | Whether to use a high-risk technology should be decided by also considering the magnitude of its benefits. | 0.794 | 0.013 |
|  | 7 | A useful technology should be used if its risks can be controlled, even if some risks remain. | 0.800 | 0.013 |
|  | 8 | Support for or opposition to a technology should be decided by weighing both its risks and benefits. | 0.661 | 0.017 |
| F3: Trade-off between different risks | 9 | If a readily apparent hazard is eliminated, the risks that emerge afterward will necessarily be smaller. | 0.571 | 0.019 |
|  | 10 | If the known risks of technologies or chemicals are eliminated, the overall risk to society will necessarily decrease. | 0.617 | 0.017 |
|  | 11 | If food additives were entirely eliminated, the risks of food would disappear. | 0.881 | 0.009 |
|  | 12 | If pesticides were entirely eliminated, the risks of food would decrease. | 0.868 | 0.009 |
| F4: Paradox of risk perception | 13 | The various risks of technologies and chemicals will continue to increase in the future. | 0.735 | 0.013 |
|  | 14 | Compared with 100 years ago, the risks in daily life and social life have increased. | 0.905 | 0.007 |
|  | 15 | Compared with 100 years ago, serious life-threatening hazards have increased in present-day Japan. | 0.898 | 0.007 |
|  | 16 | In Japan, the risks threatening the lives of infants have increased compared with 100 years ago. | 0.706 | 0.014 |
| F5: Basic knowledge of risk | 17 | I can intuitively grasp how often an event that occurs only rarely (e.g., a one-in-ten-thousand chance) actually happens. | 0.560 | 0.019 |
|  | 18 | I can explain the difference between risk and hazard. | 0.822 | 0.010 |
|  | 19 | I know the concept (definition) of risk as a probability. | 0.901 | 0.008 |
|  | 20 | I know examples of how the concept of risk is used to protect safety. | 0.875 | 0.008 |
| F6: Cognitive bias in risk perception | 21 | Novel technologies are more dangerous than long-established ones. | 0.688 | 0.015 |
|  | 22 | Even a very small amount of radiation inevitably causes serious health effects. | 0.883 | 0.008 |
|  | 23 | Even a very small amount of a carcinogenic substance entering the body inevitably causes serious health effects. | 0.908 | 0.007 |
|  | 24 | Artificial radiation, such as that from nuclear power plants, is more harmful to the human body than natural radiation of the same type and amount. | 0.770 | 0.012 |

Note. The Risk Literacy Scale was originally developed and published in Japanese (Kanazawa et al., 2020); no official English version exists. The English item content shown here was translated by the authors for reference purposes only and was not used in data collection. Reverse-coded items (F1, F3, F4, and F6) were recoded prior to analysis so that higher scores consistently indicate greater risk literacy. All standardized factor loadings were statistically significant (p < .001).

**Supplementary Table S3.** Inter-factor correlations of the Risk Literacy Scale

|  | F1 | F2 | F3 | F4 | F5 | F6 |
| --- | --- | --- | --- | --- | --- | --- |
| F1 | - |  |  |  |  |  |
| F2 | -0.122 | - |  |  |  |  |
| F3 | 0.481 | -0.370 | - |  |  |  |
| F4 | 0.637 | -0.307 | 0.439 | - |  |  |
| F5 | -0.083 | 0.385 | -0.353 | -0.188 | - |  |
| F6 | 0.579 | -0.179 | 0.631 | 0.577 | -0.319 | - |

Standardized inter-factor correlations from the confirmatory factor analysis.

**Supplementary Table S4.** Adjusted Odds Ratios for High Concern about Heritable Effects by Categorical Radiation Knowledge Score

| **Radiation knowledge score** | **n** | **OR** | **95%CI** | **P** |
| --- | --- | --- | --- | --- |
| 0 (Ref) | 331 | 1.00 | — | — |
| 1 | 588 | 1.42 | 1.05–1.91 | .022 |
| 2 | 302 | 0.92 | 0.65–1.31 | .654 |
| 3 | 236 | 0.46 | 0.30–0.69 | <.001 |
| 4 | 53 | 0.43 | 0.18–0.93 | .040 |
| 5 | 19 | 0.19 | 0.01–1.06 | .126 |

Note. N = 1,529. Values represent Odds Ratios (OR) with 95% confidence intervals in parentheses, from a model replacing the continuous knowledge score in Model 4 with knowledge entered as a categorical variable, adjusted for gender, age, residential area, educational attainment, and the six risk literacy subscales.

**Supplementary Table S5.** Region-stratified odds ratios and residence × predictor interaction terms for Model 4

| Predictor | Fukushima OR (95%CI) | Tokyo OR (95%CI) | Interaction OR (95%CI)^a^ |
| --- | --- | --- | --- |
| Knowledge | 0.71 (0.61–0.83) ^***^ | 0.83 (0.71–0.96) ^*^ | 1.18 (0.95–1.46) |
| F1: Zero-risk orientation | 0.95 (0.79–1.15) | 0.94 (0.76–1.16) | 0.94 (0.71–1.25) |
| F2: Trade-off & Benefits | 0.76 (0.61–0.93) ^**^ | 0.81 (0.65–1.01) | 1.05 (0.78–1.42) |
| F3: Trade-off & Risks | 0.98 (0.79–1.23) | 1.10 (0.86–1.41) | 1.12 (0.81–1.56) |
| F4: Cognitive Paradox | 0.68 (0.54–0.85) ^***^ | 0.73 (0.57–0.92) ^**^ | 1.04 (0.75–1.43) |
| F5: Basic Knowledge of Risk | 0.83 (0.68–1.01) | 0.73 (0.59–0.90) ^**^ | 0.90 (0.68–1.20) |
| F6: Risk Perception Biases | 0.60 (0.47–0.75) ^***^ | 0.47 (0.35–0.63) ^***^ | 0.82 (0.57–1.18) |

Note. N = 1,529 (Fukushima n = 772; Tokyo n = 757). OR (95% CI), adjusted for age, gender, and education. ᵃInteraction OR and CI are Wald-based; all other CIs are profile-likelihood. ***p < .001, **p < .01, *p < .05.

**Methods Note**

To identify which risk literacy dimensions drove the mediation effect observed for the overall risk literacy score, exploratory subscale-specific mediation analyses were conducted for the four subscales significantly associated with heritable concern in the multivariable model (F2, F4, F5, and F6; Table 3, Model 4). Each subscale was entered individually as the mediator, using the same covariate structure as the main mediation model. Given the four comparisons, a Bonferroni-corrected significance threshold of α = .0125 (.05/4) was applied.

**Supplementary Table S6.** Subscale-specific mediation analyses (Knowledge → subscale → heritable concern)

| Subscale | ACME  (95% CI) | ACME p | ADE  (95% CI) | ADE p | Proportion mediated  (95% CI) | Significant after Bonferroni correction |
| --- | --- | --- | --- | --- | --- | --- |
| F2: Trade-off between risk and benefit | -0.0002  (-0.0027,  0.0022) | .856 | -0.086  (-0.111,  -0.062) | <.001 | 0.2%  (-2.7%, 3.4%) | No |
| F4: Paradox of risk perception | -0.0059  (-0.0118,  -0.0001) | .044 | -0.082  (-0.105,  -0.059) | <.001 | 6.7%  (0.1%, 13.8%) | No |
| F5: Basic knowledge of risk | +0.0009  (-0.0012,  0.0035) | .404 | -0.088  (-0.113,  -0.063) | <.001 | -1.0%  (-4.1%, 1.3%) | No |
| F6: Cognitive bias in risk perception | -0.0227  (-0.0305,  -0.0152) | <.001 | -0.065  (-0.089,  -0.044) | <.001 | 25.9%  (16.8%, 37.6%) | Yes |

Note. ACME = Average Causal Mediation Effect; ADE = Average Direct Effect. Estimates derived from quasi-Bayesian confidence intervals with 1,000 simulations. Bonferroni-corrected significance threshold: α = .0125

**Supplementary Figure S1.** Subscale-specific mediation path diagrams

Subscale-specific causal mediation analyses (Knowledge → subscale → heritable concern) for the four risk literacy subscales significantly associated with concern in the multivariable model (Table 3, Model 4). Solid arrows indicate statistically significant paths (p < .05); dashed arrows indicate non-significant paths. Only F6 (cognitive bias in risk perception) retained statistical significance after Bonferroni correction (α = .0125), with a proportion mediated of 25.9% (95% CI [16.8%, 37.6%]). Estimates derived from quasi-Bayesian confidence intervals with 1,000 simulations (seed fixed for reproducibility). ACME = Average Causal Mediation Effect. ***p < .001 after Bonferroni correction.
